# Specific overexpression of SIRT1 in mesenchymal stem cells rescues hematopoiesis niche in BMI1 knockout mice through promoting CXCL12 expression

**DOI:** 10.7150/ijbs.63876

**Published:** 2022-02-28

**Authors:** Jinbo Li, Xing Li, Wen Sun, Jiao Zhang, Quanquan Yan, Jun Wu, Jianliang Jin, Ruinan Lu, Dengshun Miao

**Affiliations:** 1 Department of Pharmacology, Institute of Medical and Health Science, Hebei Medical University, Shijiazhuang, Hebei 050011, China; 2 State Key Laboratory of Reproductive Medicine, Nanjing Medical University, Nanjing, Jiangsu 210029, China; 3 Department of Plastic Surgery, The Affiliated Friendship Plastic Surgery Hospital of Nanjing Medical University, Nanjing, Jiangsu 210029, China; 4 Jiangsu Key Laboratory of Oral Diseases, Nanjing Medical University, Nanjing, Jiangsu 210029, China; 5 Department of Immuno-oncology, Fourth Hospital of Hebei Medical University, Shijiazhuang, Hebei 050011, China; 6 Department of Human Anatomy, Nanjing Medical University, Nanjing, Jiangsu 210029, China; 7 Department of Hematology, Jiangsu People's Hospital, Nanjing, Jiangsu 210029, China

**Keywords:** mesenchymal progenitor cells, hematopoietic stem cell niche, Bmi1, Sirt1, CXCL12.

## Abstract

Osteoblastic lineage cells (OBCs) are bone-building cells and essential component of hematopoietic niche, but mechanisms whereby bone-building and hematopoiesis-supportive activities of OBCs could be regulated simultaneously remain largely unknown. Here we found that B cell-specific Moloney murine leukemia virus integration site 1 (Bmi1) was involved in such a co-regulatory mechanism. In this study, we first found that, accompanied with marked decline of osteogenic activity, the hematopoietic niche in Bmi1 knockout (KO) mice was severely impaired and manifested as CXCL12 expression falls and LSK homing failure; however, intratibial injection with CXCL12 effectively facilitated LSK accumulation in bone marrow of Bmi1 KO mice. To try to rescue these defects in Bmi1 KO mice, we generated Bmi1^KO^/Sirt1^Tg^ (KO-TG) double mutant mice with Sirt1 specific overexpression in mesenchymal progenitor cells (MPCs) in Bmi1 KO mice, and our data showed that KO-TG mice had significantly increased bone-building activity, elevated Cxcl12 expression by MPCs, increased LSK homing and expanded LSK pool in bone marrow compared to Bmi1 KO mice. Of note, similar improvements in KO-TG mice were observed in Bmi1 KO mice fed with dietary resveratrol, an established Sirt1 activator, comparing with KO control mice. Therefore, pharmacologic activation of Bmi1/Sirt1 signaling pathway could simultaneously promote bone-building and hematopoiesis-supportive activities of OBCs.

## Introduction

Hematopoiesis is maintained by hematopoietic stem cells (HSC) that are capable of continuously self-renewal and differentiate into committed hematopoietic progenitors that finally differentiate into various blood cells 1, 2. The process of hematopoiesis is intensively regulated by cell-intrinsic and -extrinsic pathways that corporately control blood cell generation and maturation 3. HSC niche that is comprised by various types of cells and cytokines varies extensively during lifetime. Mayack et al. reported that impaired hematopoiesis in aged mice could be effectively rescued by stem cell supportive niche of young mice using heterochronic models, and that osteoblastic niche of aged mice could be rejuvenated by circulating factors from young mice 4, suggesting crucial roles of HSC niche and its potential as a target to treat hematopoietic pathogenesis 5.

B cell-specific Moloney murine leukemia virus integration site 1 (Bmi1), a polycomb group protein, is first identified in B-cell lymphoma. Bmi1 overexpression has been previously reported in various types of cancers 6. Loss of Bmi1 causes apoptosis of human cancer cells 7 and stem cells 8. Inactivation of Bmi1 in mice results in premature aging-like phenotypes 9, including axial bone loss 10 and progressive loss of hematopoietic cells 11. Bmi1 regulates HSC self-renewal through suppressing cyclin-dependent kinase (CDK) inhibitors, p16^Ink4a^/p19^Arf^, that induce cell senescence and proliferation arrest 11, 12. Bmi1 deficiency causes significant reduction of lymphoid and myeloid cell generation in mice with Bmi1 global deletion 9 or with specific deletion in hematopoietic cells 13. p16^Ink4a^/p19^Arf^ genetic depletion efficiently rescues the defects in HSC, but not in HSC niche, in Bmi1 global knockout (KO) mice 12, suggesting an important role of Bmi1 in regulating HSC niche and this regulation is not p16^Ink4a^/p19^Arf^-dependent. We reported that Bmi1 KO mice have serious bone loss associated with reduced osteoblast differentiation from mesenchymal progenitor cells (MPCs) and impaired bone-building activity 10; however, it remains unknown if Bmi1 deficiency impairs the supportive role of osteoblastic niche in HSC regulation. If so, what's the underlying mechanism of this process.

Sirtuin 1 (Sirt1), a mammalian homologue of silent information regulator 2 (Sir2), is pleiotropic nicotinamide adenine dinucleotide (NAD)-based deacetylase. Sirt1 removes acetyl groups from DNA repairing proteins, nuclear histones and transcription factors, thereby regulating transcription activity of target genes 14. Similar with biological function of calorie restriction 15, activation of Sirt1 promotes longevity 16, 17. Our published data shows that Sirt1 reduces in Bmi1 KO MPCs, and activation of Sirt1 partially rescues the defects of osteoblast differentiation from these cells 10, suggesting that Sirt1 mediates Bmi1-induced osteoblast differentiation of MPCs. Resveratrol (3,5,4'-trihydroxystilbene), a plant polyphenol, has potential to activate Sirt1 expression and extend life span of simple organisms, including yeast and nematode 18, and rodent models 19, 20; Remarkably, Zhang et al. provides that evidence that Sirt1-mimetic drug resveratrol rescues hematopoietic deficits in Fancd2 KO mice, a typical murine model of Fanconi Anemia in humans 21. Briefly, resveratrol improves the bone marrow microenvironment, maintains LSK cells in a quiescent state and increases the spleen colony-forming capacity of bone marrow cells in Fancd2 KO mice 21. However, it is unclear whether overexpression of Sirt1 in MPCs or resveratrol supplementation can rescue osteoblastic niche and hematopoiesis defects in Bmi1 KO mice.

Therefore, we hypothesized that osteoblastic niche in Bmi1 KO mice was impaired and that Sirt1 specific overexpression in mesenchymal lineage or dietary resveratrol supplementation could rescue defects in bone-building and hematopoiesis-supportive activity of MPCs in Bmi1 KO mice. To study this, bone marrow transplantation and co-culture experiments were performed to investigate the potential defects of HSC niche in Bmi1 KO mice. We next developed Sirt1 transgenic (Sirt1^Tg^) mice with Sirt1 specific overexpression in MPCs driven by Prx1 gene promotor, and further generated Bmi1/Sirt1 double mutant mice to determine whether Sirt1 overexpression in MPCs could rescue bone formation and hematopoiesis deficits in Bmi1 KO mice. Finally, Bmi1 KO mice were supplemented with dietary resveratrol to verify potential findings in Bmi1 KO mice with Sirt1 genetic overexpression.

## Methods and materials

### Animals

In this study, Bmi1 heterozygous mice were kindly provided by Dr. Anton Berns in the Netherlands Cancer Institute, and these mice were genotyped as described previously 10. Bmi1 homozygous mice develop premature aging-like phenotype and have short life span. The Sirt1 transgenic (Sirt1^TG^) mice we generated with Sirt1 specific overexpression under control of the 2.4 kb Prx1 gene promoter. PCR was conducted and DNA was amplified using the forward primer 5'-TCCCTCAAAGTAAGACCAGTAGC-3' and a reverse primer 5'-TTTCTCACTGTTCCAGCCAC-3'. Bmi1^KO^/Sirt1^TG^ double mutant mice used in this study were generated by mating Bmi1 heterozygous mice with Sirt1^TG^ mice. 4-week-old wild type (WT) and Bmi1 KO mice were fed with resveratrol diet for 3 weeks before sacrifice. This specific resveratrol diet was made by mixing powdered resveratrol (3,5,4'-trihydroxystilbene) in common rodent diet by Beijing Vital River Laboratory Animal Technology Co., and the mice were fed with this resveratrol diet at 250 mg resveratrol/kg body weight/day as described previously 21. No random allocation was performed because specific genotypes of mice were required for our studies. Five mice or fewer were housed in individual cages in a SPF (pathogen free) room and fed with a laboratory autoclaved diet (TianChi, China; #LAD2003) and potable, uncontaminated drinking water according to NIH-41 Guidelines. All of the mice were bred and maintained in SPF Laboratory Animal Center of Nanjing Medical University. The use of animals in this study was approved by the Institutional Animal Care and Use Committee of Nanjing Medical University (Approval ID 1601253).

### Histology, Histochemistry and Immunohistochemistry

Tibiae were dissected and soft tissue was removed before fixation in PLP fixative solution (2% paraformaldehyde containing 0.075 M lysine and 0.01 M sodium periodate) 48 h at 4°C. Hard tissue was decalcified and processed for histological analysis as described previously 22. The paraffin sections (5 μm of thickness) were stained with hematoxylin & eosin (H&E), and histochemically stained for alkaline phosphatase (ALP) and immunohistochemically stained for type I collagen expression as described previously 10.

### Quantitative real-time PCR

RNA was extracted from mouse long bones and mesenchymal progenitor cells using Trizol reagent (Invitrogen) according to the manufacturer's protocol. Reverse-transcription reactions and Real-time PCR were performed as described previously 23. Melting curves obtained at the end of each run were used to discriminate specific from nonspecific cDNA products. Primer sequences are as follows: Alkaline phosphatase (*Alp*), forward, 5'-CTTGCTGGTGGAAGGAGGCAGG-3', and reverse, 5'-GGAGCACAGGAAGTTGGGAC-3'; Integrin β1, forward, 5'-GAGGTCGTTCTTCAGTTCATC-3', and reverse, 5'- CCACAGACACATTCTCCATTG-3'; Integrin β2, forward, 5'-GCTCATCAAGAATGCCTAC-3', and reverse, 5'-CCTGGATACACTCGGAAG-3'; C-X-C motif chemokine ligand 12 (*Cxcl12*), forward, 5'-GCATCAGTGACGGTAAAC-3', and reverse, 5'-GCAGCCTTTCTCTTCTTC-3'; N-cadherin, forward, 5'-TCATCGCTATCCTTCTGTGTATC-3', and reverse, 5'- AGTCCTGGTCTTCTTCTCCTC-3'; Jagged-1 (*Jag1*), forward, 5'-CATAGCCTGTGAGCCTTCC-3', and reverse, 5'-GCAACCGCAGCAATAAGTG-3'; Angiopoietin-1 (*Ang1*), forward, 5'-TGCCATTCTGACTCACATAGG-3', and reverse, 5'-GCTCTGTCGCACTCTCAC-3'; Vascular cell adhesion molecule 1 (*Vcam1*), forward, 5'-TGTGGAAATGTGCCCGAAAC-3', and reverse, 5'-TTGTGAGCCAACTTCAGTCTTAG-3'; Stem cell factor (*Scf*), forward, 5'-GTGGATGACCTCGTGTTATGC-3', and reverse, 5'-CGGCTTTCCTATTACTGCTACTG-3'; Osteopontin (*Opn*), forward, 5'-TCTCAGAAGCAGAATCTCCTTG-3', and reverse, 5'-CATCCGAGTCCACAGAATCC-3'; Glyceraldehyde 3-phosphate dehydrogenase (*Gapdh*), forward, 5'-GGTCGGTGTGAACGGATTTG-3', and reverse, 5'-ATGAGCCCTTCCACAATG-3'. The methods for calculating the relative abundance of each gene followed the previous description 24.

**Western blot analysis**. Protein extracted from multiple types of tissue or cells was quantitated using a commercial kit (Bio-Rad). Homogenized multiple tissues and cells were lysed in RIPA lysis with protease inhibitor cocktail (Roche, 11836153001). 30 μg protein for each sample were fractionated by SDS-PAGE and transferred to nitrocellulose membranes. Immunoblotting was carried out as described previously 22 using anti-Sirt1 (Abcam, ab12193), -CXCL12 (Santa Cruz, sc-6193), -Ang1 (Millipore, Ab3120), -GAPDH (Santa Cruz, sc-32233) and -β-actin (Sigma, A5441) antibodies. Bands were visualized using enhanced chemiluminescence (ECL, Amersham) and quantitated by Image-Pro Plus (Media Cybernetics, USA).

### Bone marrow transplantation

Six-week-old WT, Bmi1^KO^ and Bmi1^KO^/Sirt1^Tg^ double mutant male mice were irradiated at a dose of 9.5 Gy using an RS-2000 X-ray Irradiator (Rad Source Technologies, USA). The parameters for the machine settings were 160 kV, 25 mA and a dose rate of 1.875 Gy/minute. 5×10^6^ bone marrow cells harvested from enhanced GFP transgenic mice were suspended in 100 µL α-MEM (Invitrogen, USA) and injected into irradiated recipients via the tail vein as previously described 25, 26. Two weeks after transplantation, recipients were euthanized for further analysis.

### Intratibial injection of CXCL12 protein

Recombinant mouse CXCL12/SDF-1 alpha protein (R&D, 460-SD-010) was stored at -80 °C at a concentration of 100 μg/mL in sterile PBS following the manufacturer product instruction and diluted by 500 times in 0.9% sterile saline to achieve a final working concentration of 4 ng/20 μL immediately prior to microinjection. Since the amount of CXCL12 in the bone marrow extracellular fluid from a femur of WT mice is around 400 pg 27 and the half-time of CXCL12 is less than 30 min 28, 4 ng recombinant mouse CXCL12 in 20 μL was microinjected into each tibia and mice were harvested one week post microinjection. The same volume of 0.9% sterile saline served as the vehicle group.

### Flow cytometry

For analysis of LSK cells, single-cell suspension of bone marrow and spleen cells was made and filtered using 40 μm strainer before staining. 2×10^6^ BM cells were suspended in 100 μL FACS buffer (sterile PBS buffer containing 2% fetal bovine serum) and incubated with PE-conjugated anti-Sca1 (BioLegend; #108107), PE-Cy5-conjugated anti-c-Kit (eBioscience; #15-1171-82), and FITC- (eBioscience; #22-7770-72) or APC- (BD; #558074) conjugated Mouse Lineage Antibody cocktail at 4℃ in dark for 30 min. Cells were then rinsed once with 2% FBS-contained FACS buffer and resuspended in FACS buffer for analysis. To detect Annexin V, CD44 and CXCR4 expression on LSK surfaces, APC-conjugated Annexin V (#640920) or CD44 (#103012) or CXCR4 (#146508) from BioLegend were added in staining panel with LSK surface markers and stained the cells simultaneously. To detect Ki67 expression, the bone marrow cells were first stained with LSK surface markers, and then fixed/permeabilized use Fixation/Permeabilization solution kit (BD; #554714); for intracellular staining, APC-conjugated anti-Ki67 (eBioscience; #17-5698-82) and rabbit anti-mouse NICD (Abcam; #ab8925) were applied, Alex Flour 647 conjugated goat anti-rabbit IgG (Invitrogen; #A27040) as a secondary antibody to detect NICD expression. All staining performed at 4℃ in dark according to manufacturer's instructions. Flow analyses were performed on FACSCalibur and FACSCelesta (BD Biosciences) systems. Collection of all flow cytometry data was done using BD CellQuest Pro Software. Analysis of all flow cytometry data was done using FlowJo V9 software (TreeStar) and FCS express (De Novo software).

### Conditioned medium and BM cell culture

1×10^6^ 3^rd^ passage of bone-derived mesenchymal progenitor cells (BdMPC) from WT and KO mice were seeded in each 100 mm cell culture dish and maintained in αMEM medium containing 10% FBS and 50 U/ml penicillin/streptomycin. When ~80% confluence was reached, cell culture medium was discarded and cells were gently rinsed once with PBS, then cells were incubated with serum free αMEM containing 50 U/ml penicillin/streptomycin at 37°C, 5% CO_2_ for 24 h, then cell culture supernatant was collected. For collection of conditioned mediums from KO BdMPCs treated with vehicle or resveratrol, resveratrol was added in culture medium 8 h after cell seeding and maintained for 48 h. When ~80% confluence was reached, cells were rinsed with PBS gently and changed to serum free αMEM as described above for 24 h before collecting supernatant. Bone marrow was flushed out from the leg bones of 4-week-old WT and KO littermates with αMEM containing 10% FBS using a syringe and 25-gauge needle. 5×10^6^ total bone marrow cells were suspended in 3 ml conditioned culture medium (1.5 ml collected supernatant (serum free) mixed with 1.5 ml fresh culture medium containing 30% FBS) with 20 μg/ml control IgG or 20 μg/ml anti-CXCL12 neutralizing antibody (Millipore; #MABC184) or 20 μg/ml angiopoietin blocking peptide (MyBioSource; #MBS8243845) and seeded in each well of Nunc^TM^ non-treated 6-well plate from Thermo Scientific. This bone marrow cell culture was maintained for 48 h before being collected for flow analysis.

### Plasmid transfections and cell treatment

3^rd^ passage of BdMPCs generated from leg bones of 1-month-old male C57BL6/J mice were detached from culture dishes with 1× trypsin-EDTA, and cells were spun down and resuspended in 500 μL electroporation buffer (Thermo Electron) at a concentration of 1 × 10^6^ cells per 100 μL. Mixed 10 μg of plasmids, Bmi1-pcDNA3.1 or vector, with 500 μL of cells and transferred into an electroporation cuvette at room temperature. Electroporation was done using BTX ECM 830 electroporation system, one electric pulse of 480 V/cm for 100 milliseconds. Immediately after electroporation, 500 μL complete culture medium was added in cuvette and cells were transferred into culture vessel gently. 5 × 10^4^ cells were seeded in each well of 8-well chamber slides (Thermo Scientific; #154534PK) overnight and dead cells were discarded by changing with complete culture medium (αMEM; 10% FBS). 48 h after transfection, cells were fixed with 4% paraformaldehyde at room temperature for 5 min before immunofluorescence staining. Culture medium was changed every other day.

1 x 10^6^ 3^rd^ passage of BdMPCs from 1-month-old WT and KO mice were seeded in each 100 mm cell culture dishes. When 70 - 80% confluence reached, cells were treated with 40 μM resveratrol for 24 or 48 h for extracting mRNA and protein, respectively. To induce osteoblast differentiation, 3^rd^ passage of BdMPC from 4-week-old WT and KO mice were treated in osteoblast induction medium containing β-glycerophosphate and ascorbic acid with vehicle or 40 μM resveratrol for 4 days, and then followed by fixation with 4% paraformaldehyde at room temperature for 5 min and alkaline phosphate staining. Culture medium was changed every other day.

### Micro-computed tomography imaging

Tibiae were dissected free of soft tissue and examined using micro-computed tomography (μCT) as described 10, and μCT measurements were followed the guideline for μCT assessment 29. The resolution of the µCT images is 18.2 μm.

### Data availability

All relevant data are available from the authors upon reasonable request.

### Statistics

All results are given as the mean ± S.D. Variance was similar between groups for most parameters assessed. Comparisons between two groups were analyzed using Student's unpaired two-tailed *t* test and those among 3 or more groups using one-way ANOVA analysis of variance followed by Tukey's post-hoc multiple comparisons using GraphPad Prism 5. *p*<0.05 was considered statistically significant. No data were excluded from these analyses and investigators carried out these analyses blinded to identify of the various groups.

## Results

### Bmi1 KO mice have defects in osteoblastic activity and hematopoietic niche

**Fig 1:**
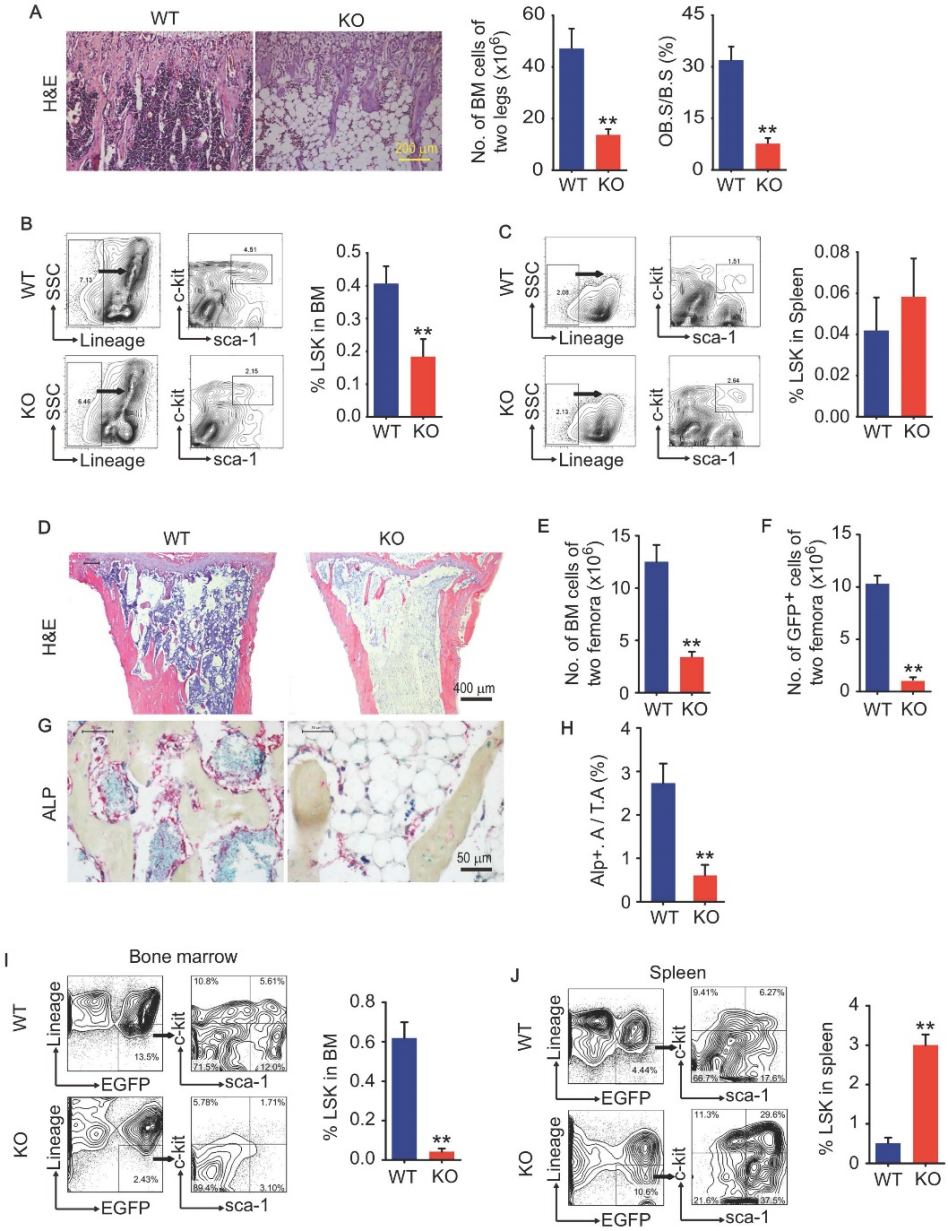
** Bmi1 deficiency causes defects in LSK homing to the bone marrow.** (A) Representative H&E-stained sections of proximal tibiae of 6-week-old WT and Bmi1 knockout (KO) male mice, and statistical analysis of bone marrow cellularity of two legs (two tibiae plus two femora), and of osteoblast surface measurement. n=5 mice/group. (B) BM cells were flushed out from femora of 6-week-old WT and KO mice and frequencies of Lineage^-^Sca1^+^cKit^+^ (LSK) cells were analyzed using flow cytometry. n=12 mice/group. (C) Frequencies of LSK cells in spleen of 6-week-old WT and KO mice measured using flow. n=5 mice/group. (D) 6-week-old WT and KO male mice were irradiated and transplanted with BM cells from 2-m-old GFP transgenic male mice. The recipient mice were sacrificed 2-week post transplantation. Representative H&E-stained sections of proximal tibiae of WT and KO mouse recipients. The numbers of total BM cells (E) and GFP^+^ BM cells (F) in BM of two femora of each WT (n=7 mice) and KO (n=4 mice) recipients were counted. (G) Representative images of ALP-stained sections of proximal tibiae showing ALP^+^ osteoblastic cells coated on trabecular bone surfaces and, and (H) histomorphometric analysis of ALP^+^ area (ALP^+^ A./T.A.) from the mice in (D-F). (I) Frequencies of donor-derived (GFP^+^) LSK cells in bone marrow of WT and KO recipients. (J) Frequencies of donor-derived (GFP^+^) LSK cells in spleen single-cell suspension analyzed as in (I). **p<0.01. Data are mean ± SD; Unpaired two-tailed Student *t* test.

We and others previously report that Bmi1 deficiency causes hematopoiesis defects that characterized by severe loss of hematopoietic stem/progenitor cells (HSPC), and defects in osteoblastic bone formation associated with limited osteoblast differentiation from MPCs. Bmi1 KO mice at 6 weeks of age had obvious accumulation of adipocytes in bone cavity and significant decrease in BM cells and osteoblasts on trabecular bone surfaces in metaphysis, comparing with WT littermates (Fig. 1A). The percentage of Lin^-^Sca1^+^cKit^+^ (LSK; HSPC) cells in BM was lower (Fig. 1B) in KO mice than WT littermates, while it was comparable in spleen between them (Fig. 1C). To explore potential defects in hematopoietic niche of KO mice, 6-week-old WT and KO mice were irradiated and transplanted with 5 × 10^6^ total BM cells from GFP transgenic mice via tail vein for each irradiated recipient. Unexpectedly, 5 in 9 (56%) KO recipients died within the first 2 weeks post BM transplantation (BMT), and only 4 KO recipients survived throughout this period, but they were weaker and less physical activity than WT recipients. We therefore sacrificed these 4 KO and all WT recipients at 2 weeks post BMT. Histomorphology analysis on H&E-stained tibial sections showed that BM cells filled up the tibial cavities of WT recipients, but not for KO recipients (Fig. 1D), indicating hematopoiesis reconstruction in KO recipients is much less efficient than that in WT recipients. The numbers of total BM cells and donor-derived GFP^+^ BM cells in femora were significantly lower in KO than WT recipients (Fig. 1E and 1F). The alkaline phosphatase (ALP)-positive osteoblasts on trabecular bone surfaces were significantly fewer in KO than WT recipients (Fig. 1G and 1H). Of note, the percentage of donor-derived GFP^+^LSK cells in spleens of KO recipients was 5-fold higher than WT recipients (Fig. 1J), while this percentage in BM of KO recipients was only around 10% of that in WT recipients (Fig. 1I), suggesting that Bmi1 expression by hematopoietic niche was required for recruiting donor-derived GFP^+^ LSK cells into BM.

### MPCs from Bmi1 KO mice fail to support hematopoietic stem/progenitor cells

**Fig 2:**
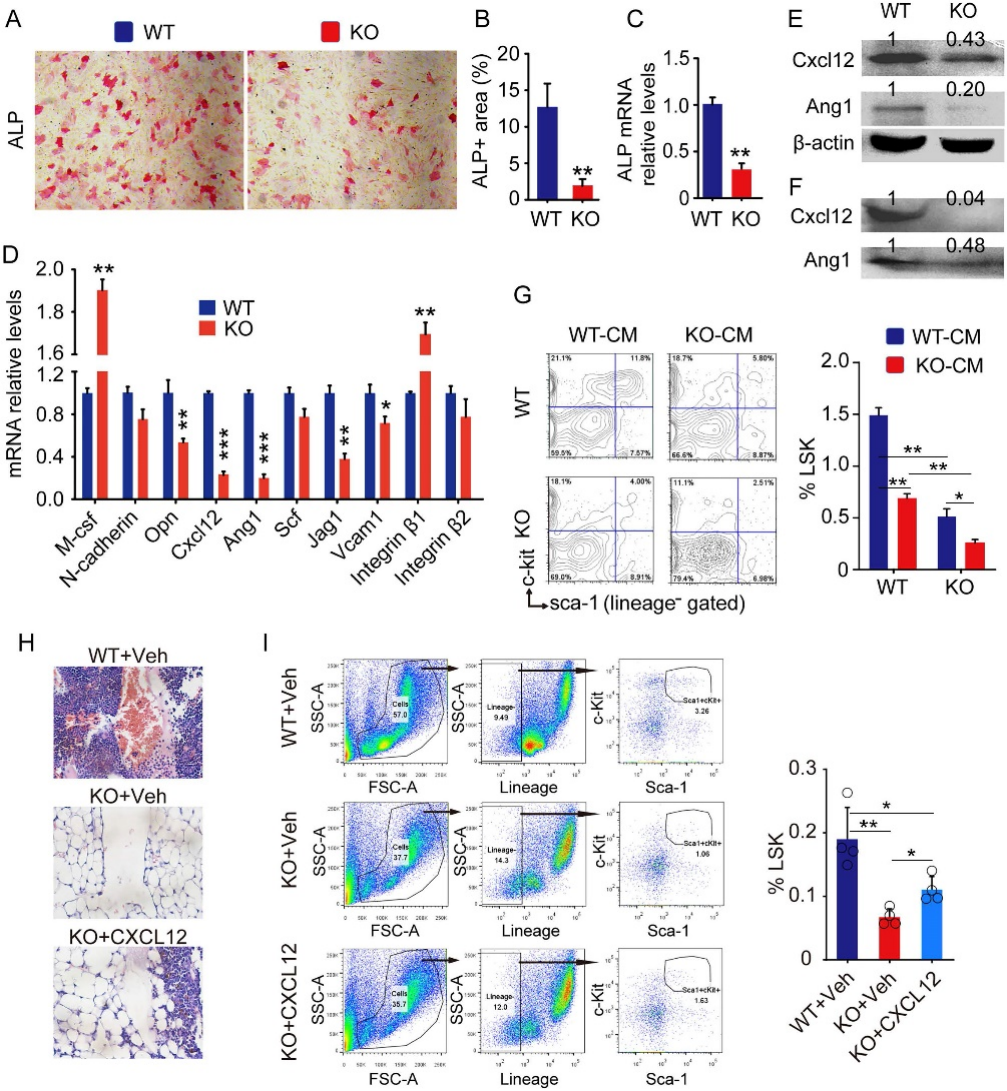
** Defects of HSPC niche composed by osteoblastic cells in Bmi1 KO mice.** (A) 3^rd^ passage of bone-derived mesenchymal progenitor cells (BdMPC) from 4-week-old WT and KO mice treated in osteoblast induction medium containing β-glycerophosphate and ascorbic acid for 4 days before ALP staining. ALP^+^ osteoblasts shown in red after ALP staining. (B) The percentage of ALP^+^ cell area versus total cell growth area was counted. n=3 samples. (C) *Alp* gene transcription levels in bulk mRNA from 3^rd^ passage of BdMPCs treated with osteoblast induction medium for 4 days in (A). n=3 samples. (D) Gene transcription levels of niche factors, including macrophage colony-stimulating factor (*M-csf*), N-cadherin, osteopontin (*Opn*), *Cxcl12*, Angiopoietin 1 (*Ang1*), stem cell factor (*Scf*), Jagged 1 (*Jag1*), *Vcam1*, *integrin β1* and *β2*, in 3^rd^ passage of BdMPCs from 3-week-old WT and KO mice were tested by qPCR. n=3 samples. (E-F) Protein levels of cellular (E) and secreted (F) CXCL12 and Ang1 were measured in cellular protein lysates and condensed medium supernatant from 3rd passage of BdMPCs by WB, respectively. (G) WT and KO BM cells were cultured in conditioned medium for 48 h before flow analysis. Conditioned medium (CM): Mixture of 50% (v/v) fresh culture medium with 50% conditioned medium collected from WT or KO BdMPC culture. The bar graph in right panel shows frequencies of LSK cells in cultured BM cells. n=4 samples. (H) Representative H&E-stained tibial paraffin sections from WT and KO mice with intratibial injection of vehicle or CXCL12. n=4 mice. (I) Flow analysis of LSK cells in BM of mice in Fig. H. *p<0.05, **p<0.01, ***p<0.001. Data are mean ± SD; Unpaired two-tailed Student *t* test in (B), (C) and (D). One-way ANOVA with Tukey's post-hoc test in (G) and (I).

To determine if Bmi1 expression by MPCs is required for maintenance of hematopoietic niche, we next characterized the bone-derived mesenchymal progenitor cells (BdMPCs) isolated from KO mice and WT littermates. We found that ALP^+^ osteoblast differentiation from KO BdMPCs was significantly lower than WT BdMPCs (Fig. 2A and 2B), and that *Alp* gene transcription levels of KO BdMPCs was significantly lower than WT BdMPCs (Fig. 2C), consistent with our published data that Bmi1 promotes osteoblast differentiation and limits adipocyte differentiation from MPCs 10. The expression of genes involved in HSPC niche regulation were next examined and the data showed that mRNA expression level of *M-csf* and *Integrin β1* by KO BdMPCs was higher than WT BdMPCs, while the mRNA level of *Opn*, *Cxcl12*, *Ang1*, *Jag1* and *Vcam1* was significantly lower in KO BdMPCs than WT BdMPCs (Fig. 2D). Among these genes, *Cxcl12* and *Ang1* mRNA expression were reduced by a greater extent than others (Fig. 2D). Consistently, expression level of Cxcl12 and Ang1 in intracellular protein (Fig. 2E) and secreted protein (Fig. 2F) from KO BdMPCs was significantly lower than WT BdMPCs. To determine the effects of potential cytokines secreted by KO BdMPCs on LSKs, co-culture experiments were performed using conditioned medium (CM) from BdMPC culture. The data showed that the percentages of LSK cells in BM cells from KO mice were significantly lower than from WT mice, when these cells were co-cultured with the same CM (Fig. 2G). Notably, the percentages of LSK cells in both WT and KO BM, when co-cultured with CM from KO BdMPCs, were significantly lower than these cells co-cultured with CM from WT BdMPCs (Fig. 2G). To test the role of CXCL12 in KO hematopoietic niche, recombinational mouse CXCL12 was intratibial injected into KO mouse tibiae and LSK populations were measured 3 days later. The results show that KO mice with CXCL12 replenishment had significantly higher percentages of LSK cells in BM than vehicle-treated KO mice (Fig.2H and 2I). These results indicated that Bmi1 deletion inhibited osteoblast differentiation from MPCs and impaired hematopoietic niche, at least partially, through downregulating CXCL12 expression.

### Sirt1 overexpression in MPCs partially rescues bone loss and CXCL12 reduction in Bmi1 KO mice

**Fig 3:**
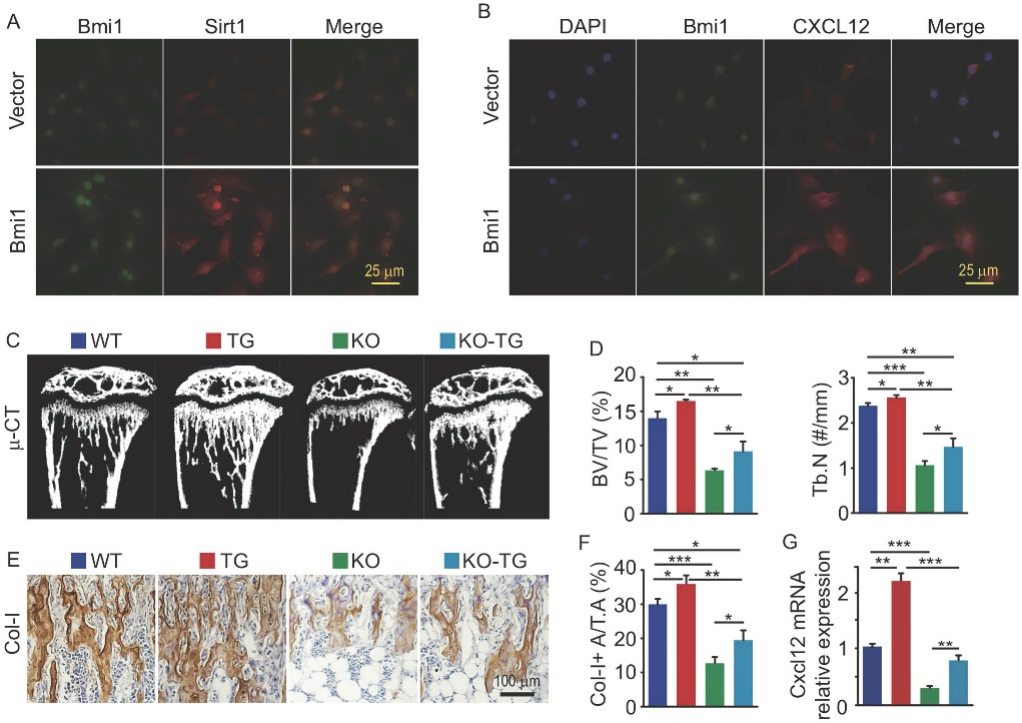
**Sirt1 overexpression in MPCs partially prevented bone loss and CXCL12 reduction in Bmi1 KO mice.** (A) BdMPCs from 1-m-old WT mice were transfected with Bmi1-pcDNA3.1 or vector plasmid using electroporation; 48 h post transfection, Bmi1 and Sirt1 expression was detected using immunofluorescence (IF) staining. (B) IF staining for Bmi1 and CXCL12 protein expression by BdMPCs as in (A). (C) Coronal sections of µCT 3D reconstruction of tibial metaphyseal region and (D) analyses of trabecular bone volume (BV/TV) and number (Tb.N) of tibiae from litters of 6-week-old WT, Sirt1 transgenic (TG), Bmi1 knockout (KO) and double mutant (KO-TG) mice. n=6 mice/group. (E) IHC staining for Collagen type-I (Col-I) expression on paraffin sections of proximal tibiae and (F) analyses of Col-1^+^ area in total tissue area (Col-I^+^ A/T.A). n=6 mice/group. (G) Cxcl12 gene transcription levels in 3^rd^ passage of BdMPCs from 6-week-old WT, TG, KO and KO-TG mice. n=3 mice/group. *p<0.05, **p<0.01, ***p<0.001. Data are mean ± SD. One-way ANOVA with Tukey's post-hoc test.

We previously reported that Sirt1 protein expression in bone tissue is lower in Bmi1 KO mice than WT mice 10. To determine if Bmi1 regulates Sirt1 expression in osteogenic cells, we transfected MPCs with pcDNA3.1-Bmi1 plasmid to overexpress Bmi1. The results of immunofluorescence staining showed that MPCs with Bmi1 overexpression had higher protein expression of Sirt1 (Fig. 3A) and CXCL12 (Fig. 3B) than vector-transfected MPCs. To next determine if Sirt1 overexpression specific in MPCs could partially rescue defects in osteogenic activity and HSPC niche in Bmi1 KO mice, we generated Sirt1 transgenic (Sirt1^Tg^) mice with Sirt1 overexpressed specifically in MPCs driven by Prx1 gene promotor and crossed them with Bmi1 heterozygous mice to generate Bmi1^KO^/Sirt1^Tg^ (so called KO-TG for short) double mutant mice. The results of µCT scanning showed that tibial trabecular bone volume and number in Sirt1^Tg^ mice was slightly higher than WT mice (Fig. 3C and 3D); Bmi1 KO mice had significantly lower trabecular bone volume and number than WT littermates, which confirmed our published data 10, while this decrease in KO mice were partially rescued in KO-TG mice (Fig. 3C and 3D). Consistent with this, the area of type I collagen^+^ (Col-I^+^) trabecular bone of tibiae from Sirt1^Tg^ mice was slightly higher than from WT mice, and the decrease of Col-I^+^ trabecular bone area in KO mice was partially rescued in KO-TG mice (Fig. 3E and 3F). Of note, Cxcl12 transcription level by BdMPCs from Sirt1^Tg^ mice was significantly higher than from WT mice; the obvious decrease in Cxcl12 transcription by BdMPCs from KO mice was effectively rescued in cells from KO-TG mice (Fig. 3G). These findings indicate that Sirt1 specific overexpression in MPCs partially prevents the loss of trabecular bone and reduction of chemokine Cxcl12 transcription in KO mice.

### Sirt1 overexpression in MPCs partially rescues the defects in osteogenic activity and hematopoiesis in Bmi1 KO mice

**Fig 4:**
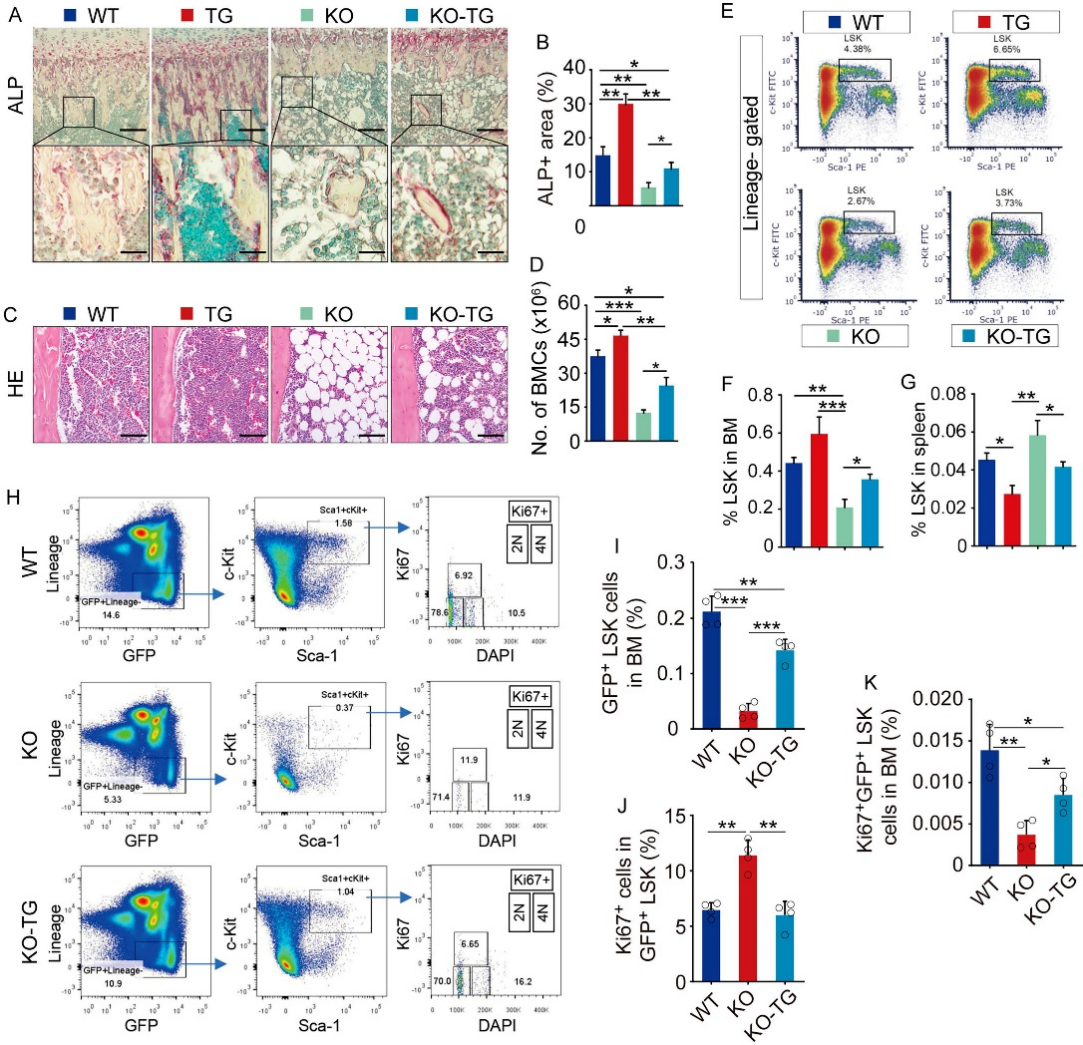
** Sirt1 overexpression in MPCs partially rescued defects of osteogenesis and hematopoiesis in Bmi1 KO mice.** (A) ALP staining on paraffin sections of proximal tibiae. Images at higher magnification (the bottom row) showed the area framed in images at lower magnification (the top row). (B) Histomorphometric analysis of ALP^+^ area in total tissue area (ALP^+^ A/T.A). n=6 mice/group. (C) Representative images of H&E-stained paraffin sections of tibiae and (D) total bone marrow cell numbers in two femora were counted. n=6 mice/group. (E) FACS gates of LSK cells in BM of mouse femora. (F-G) Flow analysis of LSK cells in BM single-cell suspension from femora (F) and spleen (G) of various mice. n=6 mice/group. (H) FACS gates of Ki67^+^GFP^+^LSK cells in BM of various recipient mice. (I-K) Statistic analysis of frequencies of GFP^+^LSK cells (I) and Ki67^+^ cells in GFP^+^LSK cells (J) and Ki67^+^GFP^+^LSKcells (K) in BM of various recipient mice. n=4 samples. *p<0.05, **p<0.01, ***p<0.001. Data are mean ± SD. One-way ANOVA with Tukey's post-hoc test.

**Fig 5:**
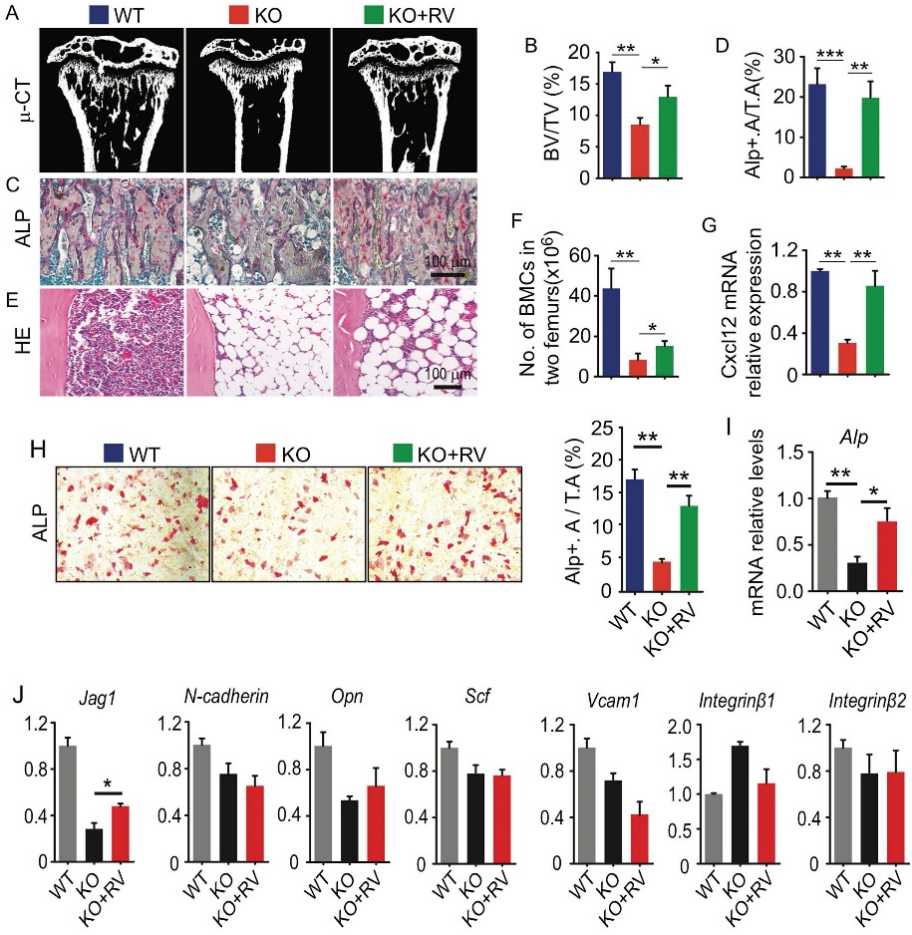
** Dietary supplementation of resveratrol partially rescues osteogenic defects in Bmi1 KO mice.** (A) Coronal sections of µCT 3D reconstruction of tibial metaphyseal region and (B) analyses of trabecular bone volume (BV/TV) in tibiae from 4-week-old WT and Bmi1 knockout (KO) mice fed with vehicle- or resveratrol (RV)-added diet for 3 weeks before sacrifice. n=6 mice/group. (C) ALP staining on paraffin sections of proximal tibiae. (D) Histomorphometric analysis of ALP^+^ area in total tissue area (ALP^+^ A/T.A). n=6 mice/group. (E) H&E-stained paraffin sections of tibiae and (F) total bone marrow cell numbers in two femora were counted. n=6 mice/group. (G) *Cxcl12* gene transcription levels in 3^rd^ passage of WT and KO BdMPCs treated with vehicle or resveratrol. n=3 samples. (H) ALP staining of 3^rd^ passage of BdMPCs from 4-week-old WT and KO mice and treated with osteoblast induction medium plus vehicle or resveratrol for 4 days. ALP^+^ cell (red) area was counted. (I) *Alp* gene transcription levels in bulk mRNA from 3^rd^ passage of WT and KO BdMPCs treated with vehicle or resveratrol. n=3 samples. (J) Gene transcription levels of niche factors, including Jagged 1 (*Jag1*), N-cadherin, osteopontin (*Opn*), stem cell factor (*Scf*), *Vcam1*, *integrin β1* and *β2*, in 3^rd^ passage of WT and KO BdMPCs treated with vehicle or resveratrol tested using qPCR. n=3 samples. *p<0.05, **p<0.01, ***p<0.001. Data are mean ± SD. One-way ANOVA with Tukey's post-hoc test.

To measure osteogenic activity, ALP staining on tibial paraffin sections from WT, Bmi1 KO, Sirt1^Tg^ (Tg) and KO-Tg mice was performed. Expectedly, ALP^+^ area on trabecular bone surfaces in metaphysis of Tg mice was significantly higher and of KO mice was significantly lower than WT mice (Fig. 4A and 4B). Notably, the reduction of ALP^+^ area in tibial metaphysis of KO mice was partially rescues in KO-Tg mice (Fig. 4A and 4B). Histomorphometric analysis of H&E-stained tibial sections revealed that Tg mice had slightly more BM cells in tibiae than WT mice, and that KO mice had significantly fewer BM cells and more adipocytes in BM cavity than WT mice (Fig. 4C). This reduction of BM cells in KO mice was effectively rescued in KO-Tg mice (Fig. 4C), which was confirmed by the data on counting total BM cell numbers in two femora from each mouse (Fig. 5D). Of note, the percentage of LSK in BM cells of Tg mice was higher than WT mice, and the LSK percentage in KO mice was lower than WT mice (Fig. 4E and 4F). This decrease in KO mice was effectively rescued in KO-Tg mice (Fig. 4E and 4F). In contrast, the percentage of LSK in spleen of Tg mice were lower than WT mice (Fig. 4G); the increase of LSK percentage in spleen of KO mice was effectively rescued in KO-Tg mice (Fig. 4G). To further determine if improvement of hematopoietic niche facilitates LSK maintenance in KO-Tg mice, whole BM cells from GFP mice were transplanted into various recipients immediately following lethal-dose radiation, the percentages of total and proliferating GFP^+^LSK in BM of recipients were measured 2 weeks post transplantation. The results show that percentages of total GFP^+^LSK in BM of KO-Tg recipients are significantly higher (Fig. 4H and 4I), but percentages of Ki67^+^ proliferating cells in total GFP^+^LSK are lower than that in KO recipients (Fig. 4H, 4J and 4K), suggesting that this increase of total GFP^+^LSK in KO-Tg recipients probably associates with elevated recruitment of LSK into BM, but not LSK proliferation. These results indicate that Sirt1 overexpression in MPCs partially rescued the osteogenic inhibition and hematopoietic niche defects in KO mice.

### Dietary supplementation of resveratrol partially rescues osteogenic defects in Bmi1 KO mice

Resveratrol (3,5,4'-trihydroxystilbene) stimulates the expression of Sirt1 and promotes osteoblast differentiation from MPCs *in vitro* 10. To determine the potential effects of resveratrol on osteogenic activity in vivo, 4-week-old Bmi1 KO mice were fed with dietary resveratrol for 3 weeks before sacrifice and the potential alterations were analyzed. We next found that trabecular bone volume (Fig. 5A and 5B) and ALP^+^ osteoblasts coating on trabecular bone surfaces (Fig. 5C and 5D) in metaphysis of tibiae of resveratrol-treated KO mice was significantly higher than vehicle-treated KO mice. BM cells in tibial diaphysis of vehicle-treated KO mice was numerically less than WT mice, while it was slightly rescued in resveratrol-treated KO mice (Fig. 5E); the number of total BM cells in two femora of resveratrol-treated KO mice was slightly, but significantly higher than vehicle-treated KO mice (Fig. 5F). We next isolated BdMPCs and induced them for osteoblast differentiation *ex vivo*. We found that inhibition of osteoblast differentiation (Fig. 5H) and Alp gene transcription (Fig. 5I) in KO MPCs was effectively rescued after resveratrol treatment. Notably, the transcription levels of niche factors, *Cxcl12 and Jag1*, in BdMPC from resveratrol-treated KO mice were higher than from vehicle-treated KO mice (Fig. 5G and 5J). These findings indicated that dietary resveratrol supplementation partially rescued the defects in bone-building activity and CXCL12 expression in Bmi1 KO mice.

### Resveratrol dietary supplement partially restores hematopoiesis in Bmi1 KO mice

**Fig 6:**
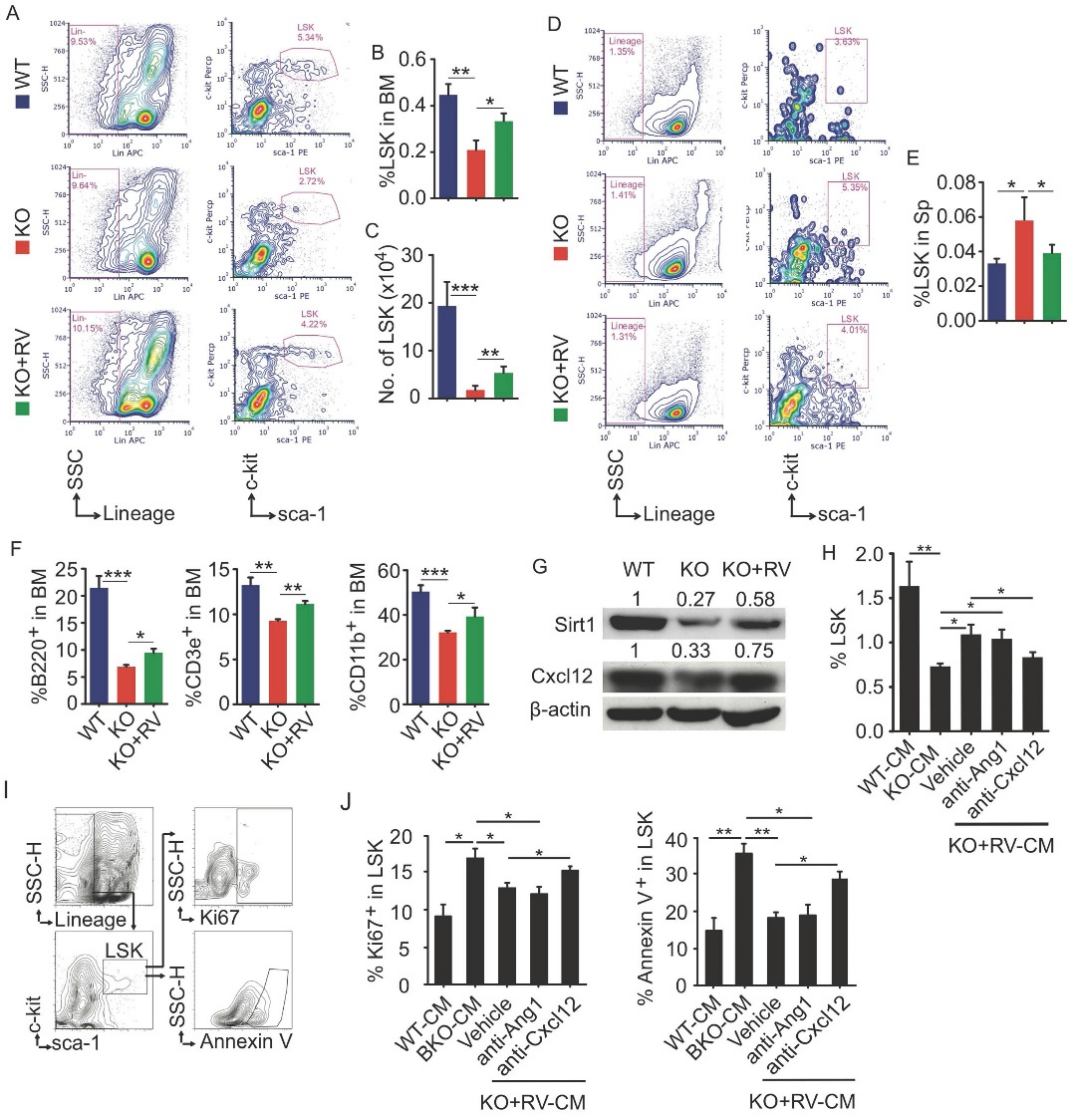
** Resveratrol dietary supplement partially restored hematopoiesis in Bmi1 KO mice.** (A) Representative flow graphs showing gates for LSK cell populations in BM of 4-week-old WT and KO mice that were fed with vehicle- or resveratrol (RV)-added diet for 3 weeks before sacrifice. (B-C) Frequencies (B) and numbers (C) of LSK in total BM cells were counted by flow. (D) Flow gates for LSK cell populations in spleen of mice as in (A) and, (E) the frequencies of LSK cells analyzed by flow. (F) The frequencies of B (B220^+^), T (CD3e^+^) and myeloid (CD11b^+^) cells in BM of mice in (A) were tested by flow. (G) WB of Sirt1, Cxcl12 and β-actin in protein lysates of WT and KO BdMPCs treated with vehicle or RV for 24 h. (H-J) BM cells from 1-m-old WT mice were cultured in conditioned medium from WT, KO and RV-treated KO BdMPCs plus Ang1 blocking peptide or anti-CXCL12 neutralizing Ab for 48 h. The frequencies of (H) LSK, (I-J) Ki67^+^ or Annexin V^+^ LSK in culture BM cells were analyzed by flow. n=4 samples. *p<0.05, **p<0.01, ***p<0.001. Data are mean ± SD. One-way ANOVA with Tukey's post-hoc test.

To further determine effects of resveratrol on HSPC in Bmi1 KO mice, flow cytometry was applied to analyze LSK pools in BM and spleen. The results showed that the percentage and the number of LSK cells in BM of KO mice were significantly lower than WT mice, while this reduction of LSK cells in KO mice was effectively rescued after resveratrol treatment (Fig. 6A-6C). In contrast, the percentage of LSK cells was higher in spleen of KO than WT control mice, while this percentage in resveratrol-fed KO mice fell down to the level of WT control mice (Fig. 6D and 6E). In addition, the percentages of B220^+^ B cells, CD3e^+^ T cells and CD11b^+^ myeloid cells in the BM were significantly lower in KO than WT control mice, while these parameters were partially rescued in resveratrol-fed KO mice, but did not reach the levels of WT control mice (Fig. 6F). We next examined the expression of Sirt1 and Cxcl12 proteins and found that their protein levels were higher in the leg bones of resveratrol-fed KO mice than in KO control mice (Fig. 6G). WT BM cells were next treated with conditioned medium (CM) harvested from WT or KO BdMPCs treated with vehicle or resveratrol. Notably, the percentage of LSK in these BM cells was significantly higher when treated with CM from resveratrol-treated KO BdMPCs than from vehicle-treated KO BdMPCs, and this increase could be abolished by CXCL12 neutralizing antibody, but not Ang1 blocking peptide (Fig. 6H). Interestingly, the percentages of Ki67^+^ proliferating LSK and Annexin V^+^ apoptotic LSK in BM cells treated with CM from KO BdMPCs were significantly higher than from WT BdMPCs (Fig. 6I and 6J); Of note, these percentages in BM cells were restored to normal levels when treated with CM from resveratrol-treated KO BdMPCs, while these beneficial effects of CM from resveratrol-treated KO BdMPCs were effectively abolished by CXCL12 neutralizing antibody, but not Ang1 blocking peptide (Fig. 6I and 6J). These results indicated that resveratrol stimulated CXCL12 expression in MPCs and hereby facilitated to LSK pool maintenance.

## Discussion

Bmi1 is critical for maintenance of HSC self-renewal capacity via suppressing p16^Ink4a^ and p19^Arf^ signaling 30, which supported by the evidence that Bmi1 KO mice develop serious pancytopenia with dramatic reduction of HSCs in BM. Hideyuki's group first observed that Bmi1 KO mice are unable to instruct hematopoietic regeneration following BM transplantation from WT donors 12. p16^Ink4a^/p19^Arf^ are established downstream targets of Bmi-1 on HSPC self-renewal regulation, while genetic depletion of p16^Ink4a^/p19^Arf^ could not rescue impaired BM microenvironment in Bmi1 KO mice 12. These previous studies suggest Bmi1 plays a critical role in the regulation of the BM microenvironment through a pathway independent of p16^Ink4a^/p19^Arf^ signaling. In this study, we have first clarified the mechanism whereby Bmi1 regulates HSC niche through positively regulating Sirt1 and chemokine CXCL12 expression, leading to enhanced osteoblastic activity and HSC maintenance.

Here we also elucidate the role of Sirt1 in the hematopoietic BM microenvironment. The protein deacetylase Sirt1 plays a broad role in anti-aging, anti-cancer, anti-inflammatory and other beneficial cardiovascular effects. We previously reported that Sirt1 was down-regulated in Bmi1 KO MPCs, and that Sirt1 overexpression and activation suppressed adipocyte differentiation from MPCs 10 and oxidative stress generation 31 in Bmi1 KO mice. Consistent with bone loss in Bmi1 KO mice, the mice with Sirt1 specific deletion in mesenchymal lineage cells driven by Prx-1 Cre 32, or in osteoblast progenitors driven by Osx-1 Cre 33, develop bone loss due to attenuated osteoblast differentiation. In this study, we found that specific overexpression of Sirt1 in mesenchymal lineage cells promoted both osteogenesis and hematopoiesis, including increased alkaline phosphate activity and trabecular bone mass, as well as expanded HSPC pool and bone marrow cellularity in Bmi1 KO mice. Thus, we propose that Sirt1 as a downstream molecule of Bmi-1 mediates the function of Bmi1 in HSPC niche regulation.

The Sirt1-mimetic and antioxidant drug resveratrol is a natural phenol that many plants produce on their own when injured or attacked by pathogens such as fungi and bacteria. The therapeutic value of resveratrol and another Sirt1 activator SRT3025 in Fanconi anemia has been established at the rodent animal level 21. Both resveratrol and SRT3025 stimulate LSK pool expansion and hematopoiesis in Fancd2 KO mice, which cannot be abrogated by conditional deletion of Sirt1 in hematopoietic cells 34, suggesting that these activators function independently of Sirt1 expression in hematopoietic stem cells. We found that conditional overexpression of Sirt1 in mesenchymal lineage cells effectively increased osteoblastic activity, HSPC pool and hematopoiesis compared to WT mice, and that Sirt1 conditional overexpression partially rescued the defects in osteoblastic bone formation and hematopoiesis in Bmi1 KO mice. These corrective effects of Sirt1 conditional overexpression in Bmi1 KO mice were mimicked by dietary supplement of resveratrol. However, the beneficial effects of resveratrol in humans remain controversial. The poor water solubility, rapid elimination (short half-life) and low bioavailability of resveratrol are the main obstacles to making it useful in clinical trials 35.

Ang1/Tie2 36 and CXCL12/CXCR4 26 are critical pathways for HSPC recruitment and maintenance. Blockade of CXCL12 signaling reduces HSPC quiescence 26 and promotes HSPC mobilization 37, 38. Greenbaum, A., et al. reported that CXCL12 produced by MPCs (driven by Prx1 Cre), but not by endothelial cells (driven by Tie2 Cre) or osteoblastic progenitors (driven by Osteorix Cre) or mature osteoblasts (driven by Osteocalcin Cre), is required for HSC maintenance 27. We found that mRNA and protein expression of Ang1 and CXCL12 in MPCs was lower in Bmi1 KO than WT mice, and that CXCL12 expression in MPCs from Bmi1 KO mice is partially restored by Sirt1 conditional overexpression or by resveratrol dietary supplementation. However, no difference in expression of CXCR4, the known receptor of CXCL12, on HSPC cell surfaces was detected between resveratrol-fed Bmi1 KO mice and KO control mice supplemental Fig. 1A and 1B). These findings indicate that Sirt1 overexpression and resveratrol dietary supplementation promotes CXCL12 expression by MSCs, rather than changing CXCR4 expression on HSPCs. Kojima, K., et al. reported that p53 activation inhibits *Cxcl12* mRNA and protein expression by bone marrow stromal cells through HIF-1α (39. The deacetylase Sirt1 inhibits the activity of p53 by removing acetylated modification of p53, suggesting that Sirt1 overexpression and resveratrol supplementation may promote the expression of CXCL12 through inhibiting p53 activation. Further study is required for this point.

Bmi1 global knockout mouse line provides an excellent animal model to study the role of Bmi1 in stem cell renewal and niche maintenance. However, these mice are susceptible to radiation and have a short life span, limiting long-term studies of bone marrow transplantation. It would be preferential if, in future studies, mice with specific deletion of Bmi1 in the Prx1Cre-driven mesenchymal lineage were generated as recipients for BM transplantation.

In this study, we first investigated the mechanism whereby Bmi1 plays a crucial role in maintaining the HSPC niche by regulating Sirt1 and CXCL12 expression. Given the current understanding that Bmi1 controls HSPC proliferation through a p16^Ink4a^/p19^Arf^ dependent pathway, our study allows us to better understand the role of Bmi1 in regulating HSPC itself and its niche through p16^Ink4a^/p19^Arf^ dependent and independent pathways, respectively.

## Supplementary Material

Supplementary figure.

## References

[1] Bryder D, Rossi DJ, Weissman IL (2006). Hematopoietic stem cells: the paradigmatic tissue-specific stem cell. Am J Pathol.

[2] Latchney SE, Calvi LM (2017). The aging hematopoietic stem cell niche: Phenotypic and functional changes and mechanisms that contribute to hematopoietic aging. Semin Hematol.

[3] Pang WW, Price EA, Sahoo D, Beerman I, Maloney WJ, Rossi DJ (2011). Human bone marrow hematopoietic stem cells are increased in frequency and myeloid-biased with age. Proc Natl Acad Sci U S A.

[4] Mayack SR, Shadrach JL, Kim FS, Wagers AJ (2010). Systemic signals regulate ageing and rejuvenation of blood stem cell niches. Nature.

[5] Conboy IM, Conboy MJ, Wagers AJ, Girma ER, Weissman IL, Rando TA (2005). Rejuvenation of aged progenitor cells by exposure to a young systemic environment. Nature.

[6] Leung C, Lingbeek M, Shakhova O, Liu J, Tanger E, Saremaslani P (2004). Bmi1 is essential for cerebellar development and is overexpressed in human medulloblastomas. Nature.

[7] Liu L, Andrews LG, Tollefsbol TO (2006). Loss of the human polycomb group protein BMI1 promotes cancer-specific cell death. Oncogene.

[8] Rizo A, Olthof S, Han L, Vellenga E, de Haan G, Schuringa JJ (2009). Repression of BMI1 in normal and leukemic human CD34(+) cells impairs self-renewal and induces apoptosis. Blood.

[9] van der Lugt NM, Domen J, Linders K, van Roon M, Robanus-Maandag E, te Riele H (1994). Posterior transformation, neurological abnormalities, and severe hematopoietic defects in mice with a targeted deletion of the bmi-1 proto-oncogene. Genes Dev.

[10] Zhang HW, Ding J, Jin JL, Guo J, Liu JN, Karaplis A (2010). Defects in mesenchymal stem cell self-renewal and cell fate determination lead to an osteopenic phenotype in Bmi-1 null mice. J Bone Miner Res.

[11] Park IK, Qian D, Kiel M, Becker MW, Pihalja M, Weissman IL (2003). Bmi-1 is required for maintenance of adult self-renewing haematopoietic stem cells. Nature.

[12] Oguro H, Iwama A, Morita Y, Kamijo T, van Lohuizen M, Nakauchi H (2006). Differential impact of Ink4a and Arf on hematopoietic stem cells and their bone marrow microenvironment in Bmi1-deficient mice. J Exp Med.

[13] Arranz L, Herrera-Merchan A, Ligos JM, de Molina A, Dominguez O, Gonzalez S (2012). Bmi1 is critical to prevent Ikaros-mediated lymphoid priming in hematopoietic stem cells. Cell cycle.

[14] Imai S, Armstrong CM, Kaeberlein M, Guarente L (2000). Transcriptional silencing and longevity protein Sir2 is an NAD-dependent histone deacetylase. Nature.

[15] Wood JG, Rogina B, Lavu S, Howitz K, Helfand SL, Tatar M (2004). Sirtuin activators mimic caloric restriction and delay ageing in metazoans. Nature.

[16] Burnett C, Valentini S, Cabreiro F, Goss M, Somogyvari M, Piper MD (2011). Absence of effects of Sir2 overexpression on lifespan in C. elegans and Drosophila. Nature.

[17] Whitaker R, Faulkner S, Miyokawa R, Burhenn L, Henriksen M, Wood JG (2013). Increased expression of Drosophila Sir2 extends life span in a dose-dependent manner. Aging (Albany NY).

[18] Hector KL, Lagisz M, Nakagawa S (2012). The effect of resveratrol on longevity across species: a meta-analysis. Biol Lett.

[19] Baur JA, Sinclair DA (2006). Therapeutic potential of resveratrol: the in vivo evidence. Nat Rev Drug Discov.

[20] Baur JA, Pearson KJ, Price NL, Jamieson HA, Lerin C, Kalra A (2006). Resveratrol improves health and survival of mice on a high-calorie diet. Nature.

[21] Zhang QS, Marquez-Loza L, Eaton L, Duncan AW, Goldman DC, Anur P (2010). Fancd2-/- mice have hematopoietic defects that can be partially corrected by resveratrol. Blood.

[22] Lu R, Wang Q, Han Y, Li J, Yang XJ, Miao D (2014). Parathyroid hormone administration improves bone marrow microenvironment and partially rescues haematopoietic defects in Bmi1-null mice. PLoS One.

[23] Zhou X, Dai X, Wu X, Ji J, Karaplis A, Goltzman D (2016). Overexpression of Bmi1 in Lymphocytes Stimulates Skeletogenesis by Improving the Osteogenic Microenvironment. Sci Rep.

[24] Li X, Sun W, Li J, Wang M, Zhang H, Pei L (2017). Clomipramine causes osteoporosis by promoting osteoclastogenesis via E3 ligase Itch, which is prevented by Zoledronic acid. Sci Rep.

[25] Li J, Cai H, Jin J, Wang Q, Miao D (2012). X-ray irradiation selectively kills thymocytes of different stages and impairs the maturation of donor-derived CD4(+)CD8(+) thymocytes in recipient thymus. J Biomed Res.

[26] Tzeng YS, Li H, Kang YL, Chen WC, Cheng WC, Lai DM (2011). Loss of Cxcl12/Sdf-1 in adult mice decreases the quiescent state of hematopoietic stem/progenitor cells and alters the pattern of hematopoietic regeneration after myelosuppression. Blood.

[27] Greenbaum A, Hsu YM, Day RB, Schuettpelz LG, Christopher MJ, Borgerding JN (2013). CXCL12 in early mesenchymal progenitors is required for haematopoietic stem-cell maintenance. Nature.

[28] Misra P, Lebeche D, Ly H, Schwarzkopf M, Diaz G, Hajjar RJ (2008). Quantitation of CXCR4 expression in myocardial infarction using 99mTc-labeled SDF-1alpha. J Nucl Med.

[29] Bouxsein ML, Boyd SK, Christiansen BA, Guldberg RE, Jepsen KJ, Muller R (2010). Guidelines for assessment of bone microstructure in rodents using micro-computed tomography. J Bone Miner Res.

[30] Klauke K, Radulovic V, Broekhuis M, Weersing E, Zwart E, Olthof S (2013). Polycomb Cbx family members mediate the balance between haematopoietic stem cell self-renewal and differentiation. Nat Cell Biol.

[31] Sun W, Qiao W, Zhou B, Hu Z, Yan Q, Wu J (2018). Overexpression of Sirt1 in mesenchymal stem cells protects against bone loss in mice by FOXO3a deacetylation and oxidative stress inhibition. Metabolism.

[32] Simic P, Zainabadi K, Bell E, Sykes DB, Saez B, Lotinun S (2013). SIRT1 regulates differentiation of mesenchymal stem cells by deacetylating beta-catenin. EMBO molecular medicine.

[33] Iyer S, Han L, Bartell SM, Kim HN, Gubrij I, de Cabo R (2014). Sirtuin1 (Sirt1) promotes cortical bone formation by preventing beta-catenin sequestration by FoxO transcription factors in osteoblast progenitors. The Journal of biological chemistry.

[34] Zhang QS, Deater M, Schubert K, Marquez-Loza L, Pelz C, Sinclair DA (2015). The Sirt1 activator SRT3025 expands hematopoietic stem and progenitor cells and improves hematopoiesis in Fanconi anemia mice. Stem Cell Res.

[35] Andrade S, Ramalho MJ, Pereira MDC, Loureiro JA (2018). Resveratrol Brain Delivery for Neurological Disorders Prevention and Treatment. Front Pharmacol.

[36] Arai F, Hirao A, Ohmura M, Sato H, Matsuoka S, Takubo K (2004). Tie2/angiopoietin-1 signaling regulates hematopoietic stem cell quiescence in the bone marrow niche. Cell.

[37] Pusic I, DiPersio JF (2010). Update on clinical experience with AMD3100, an SDF-1/CXCL12-CXCR4 inhibitor, in mobilization of hematopoietic stem and progenitor cells. Current opinion in hematology.

[38] Broxmeyer HE, Orschell CM, Clapp DW, Hangoc G, Cooper S, Plett PA (2005). Rapid mobilization of murine and human hematopoietic stem and progenitor cells with AMD3100, a CXCR4 antagonist. J Exp Med.

[39] Kojima K, McQueen T, Chen Y, Jacamo R, Konopleva M, Shinojima N (2011). p53 activation of mesenchymal stromal cells partially abrogates microenvironment-mediated resistance to FLT3 inhibition in AML through HIF-1alpha-mediated down-regulation of CXCL12. Blood.

