# Supplementary material for: Specific overexpression of SIRT1 in mesenchymal stem cells rescues hematopoiesis niche in BMI1 knockout mice through promoting CXCL12 expression
Source: Int J Biol Sci. 2022 Feb 28;18(5):2091–103. doi: 10.7150/ijbs.63876 (PMC8935221; doi:10.7150/ijbs.63876)
Supplement: Supplementary file 1 — Supplementary figure. [file ijbsv18p2091s1.pdf]

Supplemental Fig. 1

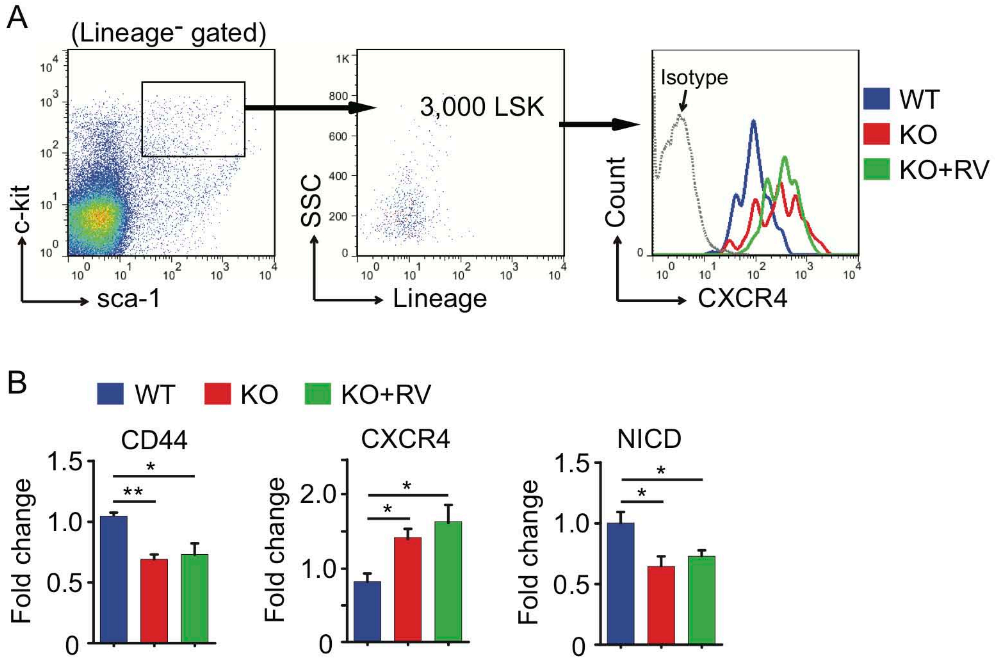

Supplemental data:

Supplemental Fig. 1 No effects of resveratrol on CD44, CXCR4 and NICD expression by LSK cells from Bmi1$^{-/-}$ mice. (A) 4-week-old WT and KO mice were fed with regular or resveratrol-added diet for 3 weeks before sacrifice for flow analysis. 3,000 LSK cells in BM of these mice were gated out for further analysis of chemokine receptor CXCR4 expression. (B) Fold changes of mean of fluorescence intensity (MFI) of CD44 and CXCR4 expression, and of Notch intracellular domain (NICD) expression by LSK cells from the mice in (A). n=6 mice/group. *p<0.05, **p<0.01. Data are mean ± SD. One-way ANOVA with Tukey's post-hoc test.
